# Sprint Interval Training Induces A Sexual Dimorphism but does not Improve Peak Bone Mass in Young and Healthy Mice

**DOI:** 10.1038/srep44047

**Published:** 2017-03-17

**Authors:** Kathrin Koenen, Isabell Knepper, Madlen Klodt, Anja Osterberg, Ioannis Stratos, Thomas Mittlmeier, Tina Histing, Michael D. Menger, Brigitte Vollmar, Sven Bruhn, Brigitte Müller-Hilke

**Affiliations:** 1 Institute for Immunology, Rostock University Medical Center, Germany; 2 Department for Trauma, Hand and Reconstructive Surgery, Rostock University Medical Center, Germany; 3 Institute for Clinical and Experimental Surgery, University of Saarland, Homburg, Saar, Germany; 4 Institute for Experimental Surgery, Rostock University Medical Center, Germany; 5 Department of Exercise Sciences, Rostock University, Germany

## Abstract

Elevated peak bone mass in early adulthood reduces the risk for osteoporotic fractures at old age. As sports participation has been correlated with elevated peak bone masses, we aimed to establish a training program that would efficiently stimulate bone accrual in healthy young mice. We combined voluntary treadmill running with sprint interval training modalities that were tailored to the individual performance limits and were of either high or intermediate intensity. Adolescent male and female STR/ort mice underwent 8 weeks of training before the hind legs were analyzed for cortical and trabecular bone parameters and biomechanical strength. Sprint interval training led to increased running speeds, confirming an efficient training. However, males and females responded differently. The males improved their running speeds in response to intermediate intensities only and accrued cortical bone at the expense of mechanical strength. High training intensities induced a significant loss of trabecular bone. The female bones showed neither adverse nor beneficial effects in response to either training intensities. Speculations about the failure to improve geometric alongside mechanical bone properties include the possibility that our training lacked sufficient axial loading, that high cardio-vascular strains adversely affect bone growth and that there are physiological limits to bone accrual.

Bone mass during the course of a life-time is constantly changing. There is growth related accrual of bone mass during foetal development, childhood and puberty that will reach its peak during the second and third decade of human life^1^,^2^,^3^. Thereafter, bone mass is continually on the decline as we age and may lead to osteopenia and osteoporosis - one of the most important musculoskeletal disorders and among the leading causes of chronic pain, frailty and loss of quality of life worldwide^4^,^5^. While there are genetic predispositions and disease related conditions that promote the development of osteoporosis, an elevated peak bone mass at the height of skeletal maturity seems to be predictive of a low risk for osteoporotic fractures at old age^1^,^6^,^7^,^8^,^9^.

As of today, it is well accepted that peak bone mass is not only determined by genetic factors and sex hormones, but also by nutrition and sports participation^2^,^3^. However, despite the general acceptance that exercise has favorable effects on bone mass, the most efficient training program and optimal type of sport for bone health are still unknown^10^,^11^. Young male and female athletes who participate in sports that involve high-impact (e.g. hurdling, karate, volleyball) or odd-impact loading (e.g. soccer, racquet games) exhibit the greatest gains in bone mass. Interestingly, swimming and cycling that do require large muscle forces yet do not provide axial loading are not associated with increased bone mass. Neither are repetitive low-impact sports such as long distance running^1^. And even though the concept of axial load shaping the bone mass is very convincing, these previous studies in humans were cross-sectional and do not provide any information on the initial bone mass at the commencement of a specific training program. Cause and consequences are therefore difficult to delineate for humans.

In order to perform longitudinal analyses on a homogeneous genetic background and to compare different training modalities for their impact on the peak bone mass during early adulthood, we here turned to a mouse model. We opted for the treadmill as training device since previous publications reported improved geometric and mechanical bone properties following moderate treadmill training in young and middle aged mice^12^,^13^. We were interested whether by increasing the cardiovascular strain and adding more axial load we would be able to not only improve running performance but also maximize peak bone mass. In order to induce a cardiovascular strain, we designed intermediate and high intensity sprint interval trainings that were tailored to 60 or 80% of the individual performance limits. An increase in axial loading was achieved via tilting the treadmill towards a 10° decline. The opposite, tilting the treadmill towards a 10° incline was supposed to add an extra strain on the muscle force. Lastly, we here decided on the STR/ort mouse strain for two reasons, (i) they have a relatively high bone mass that has not yet been analyzed for the impact of treadmill exercise and (ii) males spontaneously develop osteoarthritis later in life^14^,^15^,^16^. The intended use of these mice in the future will therefore include not only the analysis of the bone’s response to strain at advanced age, but also how exercise and changes to the bone impact on the onset and outcome of osteoarthritis. Several hypotheses prompted the present study: (i) exercise in the form of treadmill running increases bone accrual in the young and healthy, (ii) increasing either axial load or muscle force will add an extra stimulus for bone accrual, (iii) a sprint interval training that is tailored to the individual performance limit and induces a high cardiovascular strain will lead to a significantly increased bone mass that iv) can be maintained at a reduced training frequency later on. Our results are surprising in that they suggest not only a sexual dimorphism in the response to the sprint interval training but also adverse effects of high intensity treadmill training on the peak bone mass in young and healthy males.

## Results

### Sprint interval training impacted differently on the various bone parameters of male and female mice but did not improve bone strength

**Figure 1:**
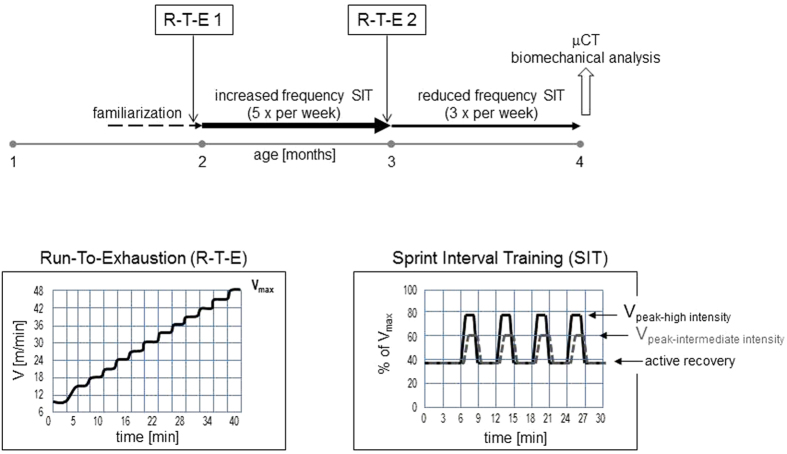
Training Programs. Treadmill training consisted of two weeks of familiarization followed by 4 weeks of increased frequency sprint interval training (five times per week) and another four weeks of reduced frequency sprint interval training (three times per week). The time line is depicted by horizontal arrows. At the end of the familiarization phase and again at the end of the four weeks of increased frequency sprint interval training, all mice took a run-to-exhaustion test in order to determine their individual performance limit. After completion of the eight weeks of sprint interval training at the age of four months, mice were sacrificed and bones of the left hind limbs were prepared for micro-computed tomography (μCT), the right femora were prepared for three-point-bending assays. Mice were weighed at entry into the familiarization phase and every week thereafter. Inserts illustrate the ramp-up run-to-exhaustion test yielding the maximal velocity (V_max_) each mouse was capable of running (left insert) and the sprint interval training with its peak velocities (V_peak_) for either high or intermediate intensity as well as active recovery phases (right insert).

We hypothesized that a highly strenuous interval training combined with an increased axial loading would lead to improved bone parameters. To test our hypothesis, we designed a treadmill training that would allow for the assessment of maximal running speeds and the adaptation of training intensities to these individual performance limits (Fig. 1). Aiming at 80% of the maximal running speed at peak velocity and tilting the treadmill downwards combined the highest cardiovascular intensity with an increased axial load. In contrast, tilting the treadmill upwards added to the muscular force required for keeping up with the speed and keeping the treadmill at an even angle served as control as did mice which did not undergo any training at all. Group sizes were N = 8 for the three training regimen and N = 9 for the non-trained controls. In order to investigate whether these sprint interval trainings led to bone accrual and improved bone strength, we here performed μCT and biomechanical analyses of the hind extremities. To this end, mice were sacrificed after having completed eight weeks of sprint interval training and were compared to the age matched non-trained controls. MicroCT analysis focused on distal femoral metaphyses for the evaluation of trabecular bone and on femoral mid-diaphyses for the evaluation of cortical bone parameters. All the parameters assessed are summarized in the Supplemental Table 1.

**Figure 2:**
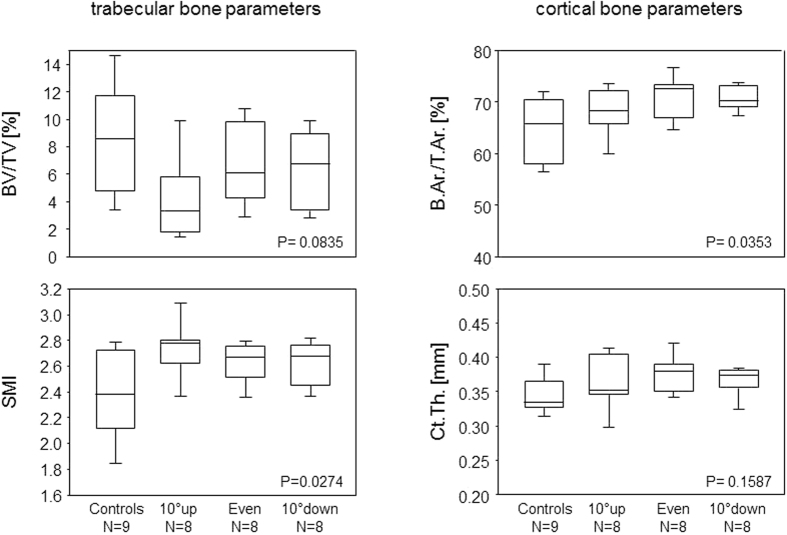
High intensity sprint interval training led to reduced trabecular and increased cortical bone mass in male mice. Box plots show trabecular and cortical bone parameters of the femora of male mice after eight weeks of high intensity sprint interval training. 10° up, even and 10° down indicate the inclination of the treadmill. P-values give the results of One-Way ANOVA. P-values < 0.05 are considered significant. The boxes represent medians as well as upper and lower quartiles and the whiskers indicate 90^th^ and 10^th^ percentiles, respectively. BV/TV: bone volume/tissue volume; SMI: structure model index: B.Ar./T.Ar.: bone area/tissue area; Ct.Th.: cortical thickness.

**Figure 3:**
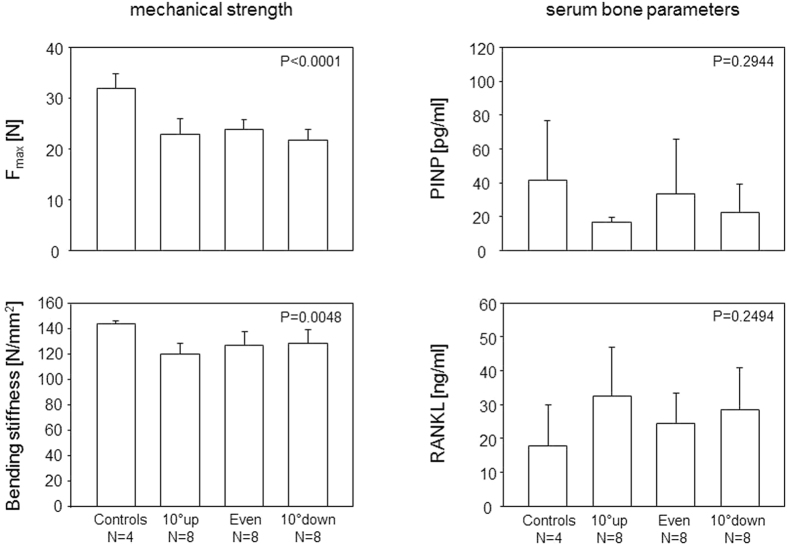
Increased femoral cortical bone mass in male mice having completed high intensity sprint interval training was paralleled by reduced mechanical strength. Bars show the means and SEM for the ultimate strength required to break the right femora and for the bending stiffness (left panels) and serum PINP and RANKL concentrations (right panels). 10° up, even and 10° down indicate the inclination of the treadmill. P-values give the results of One-Way ANOVA. P-values < 0.05 are considered significant.

Contrary to expectations, training of male mice at high intensity did not improve the trabecular bone volume (BV/TV), independent of whether the mice ran uphill, downhill or without any inclination. Moreover, the structure model index increased towards an osteoporotic phenotype, again independent of any inclination. As for the cortical bone, interval training had no impact on the cortical thickness at femoral diaphysis yet led to a significant increase of cortical bone volume (B.Ar./T.Ar) that could be attributed to a decrease in the cross-sectional tissue area (T.Ar.) (Fig. 2 and Table 1). These morphometric changes were paralleled by a decrease in mechanical strength as shown by a reduction of the ultimate strength and bending stiffness. Investigating the serum for biomarkers that would indicate a training related stimulation of bone formation or resorption, we did not observe any alterations in serum PINP and RANKL, respectively (Fig. 3).

We next tested whether the lack of bone accrual and the decreased mechanical strength following high intensity interval training in male mice was sex and intensity-specific. We therefore trained age-matched female mice at high intensity (N = 6) and introduced for both sexes an intermediate intensity training aiming at 60% of the maximal running speed at peak velocity (N = 6 for the males, N = 4 for the females). As tilting the treadmill had not yielded any significant differences, we omitted any inclination in further experiments yet for comparisons presented all mice that had trained at high intensity as one group. Moreover, in order to not miss an effect on the smaller bones closer to the actual impact, we also analyzed the tibiae.

Tables 1 and 2 present our results. They confirm that female STR/ort mice possess higher cortical and trabecular bone masses than their male counterparts^16^. Importantly, our results also show that the male femora seemed to benefit to some extent from a sprint interval training at intermediate intensity. While there was a trend towards an increased trabecular bone mass (BV/TV, Tb.Th and Tb.N.) concomitant with a reduced structure model index (SMI), the effects for the cortical bone (B.Ar/T.Ar. and Ct.Th.) were significant. As for the male tibiae, there was no effect of the sprint interval training on the trabecular bone while the cortical bone again suffered significant losses from the high intensity training (Tables 1 and 2).

Interestingly, the females’ trabecular bone did not respond to the sprint interval training at all, neither in the femora nor the tibiae and independent of the training intensity (Tables 1 and 2). Likewise, we did not observe any effect of the training on the cortical bone of the tibiae, even though our data are on the intermediate intensity training only, as we do not have data available on the high intensity training. We did though observe a trend towards increased cortical thickness in the female femora, even though the correction for multiple comparisons abolished the significance. However, performing a two-way ANOVA revealed an interaction between training and sex for the impact on the femoral cortical thickness, suggesting a sexual dimorphism (Table 1).

### Sprint interval training promoted normal physiological development

**Figure 4:**
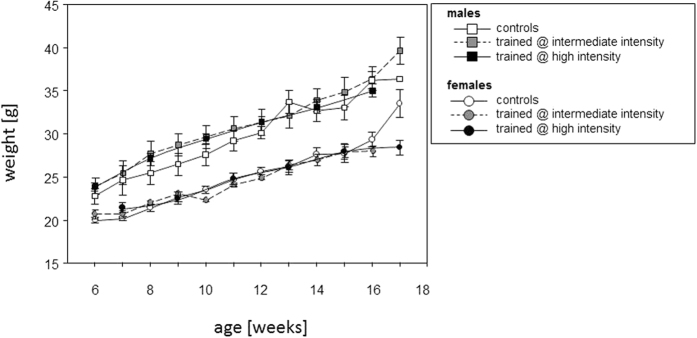
Sprint interval training promoted normal physiological development. Figure 4 shows the weight curves of mice that underwent treadmill training (grey symbols for the intermediate intensity trained mice, black symbols for the high intensity trained). The weights of non-trained age- and sex-matched control mice are interspersed (open symbols). Means and SEM are presented. Note, that mice entered the experiment at either 6 or seven weeks of age, so that experiments were terminated at either 16 or 17 weeks of age.

In order to rule out that the sprint interval training led to a deviation from the physiological development, all mice were weighed at entry into the familiarization phase and every week thereafter. Comparison to age and sex matched non-trained controls confirmed that the trained mice developed normally and gained weight like the controls. It was only during the last week of training, that the increase in weight observed for non-trained controls decelerated for the trained females (Fig. 4). Trained and non-trained controls did not show any overt differences in stature.

### Sprint interval training led to increased running speeds

**Figure 5:**
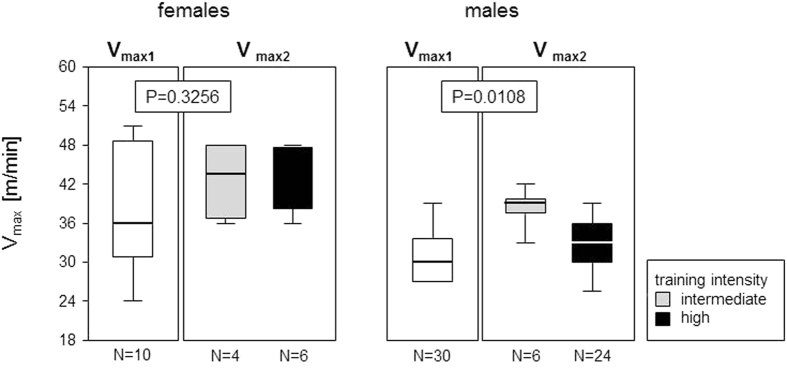
Sprint interval training led to increased running speeds. Box plots show the maximal velocities (V_max_) that mice ran voluntarily during the ramp-up run-to-exhaustion tests (R-T-E). White boxes represent the V_max1_ before commencement of any sprint interval training. Grey (intermediate intensity) and black (high intensity) boxes represent the V_max2_ after completion of four weeks of increased frequency training. The boxes represent medians as well as upper and lower quartiles and the whiskers indicate 90^th^ and 10^th^ percentiles, respectively. **Indicate P values < 0.01 resulting from Mann-Whitney U tests.

The second run-to-exhaustion test (R-T-E 2) served to assess whether four weeks of increased frequency - at either intermediate or high intensity training - led to an improved running performance of the mice. Female mice at all times ran faster than the males and increased their maximal running speeds (V_max_) from a median 36 m/min during the first R-T-E to medians of 43.8 m/min after training at intermediate and to 48 m/min after training at high intensity. However, these differences between R-T-E1 and R-T-E2 did not reach statistical significance (Fig. 5). The males’ response to the interval training was markedly different. Training at intermediate intensity significantly increased their running speeds from a median 30 to a median 39 m/min while training at high intensity showed hardly any effect (Fig. 5).

## Discussion

Our results are surprising in that they show a sexual dimorphism in young and healthy mice in response to sprint interval training. We here compared intermediate to high intensity trainings that were tailored to the individual performance limits and included peak velocities that corresponded to 60 and 80% of the maximal speeds the mice were capable of running. The males seemed to benefit most from the intermediate training intensities, in terms of improved running speed and showed adverse effects on the bone from the high intensity training. In contrast, the females’ response to the training seemed attenuated yet was most pronounced to the high intensities, both in improved running speeds and cortical bone parameters. Sprint interval training in females presented no adverse effects at all. As females possess the higher bone mass to start with (Tables 1 and 2), we do not believe that it is the bone mass per se that predisposes for a lack of response to the training. Collectively our data confirm a training effect as the mice improved their running speeds. However, our data also disprove our first hypothesis, that exercise in the form of treadmill running inevitably increases bone accrual in the young and healthy.

While our data are counterintuitive at first, they are supported by previous publications. Wallace and coworkers for instance showed exercise induced changes to the cortical bone for male C57BL/6 mice only^12^. Of note, their mice ran at a constant speed of 12 m/min only, which corresponded to the speed our males ran during their active recovery periods. However, these data are in line with our results, as they suggest that lower training intensities are beneficial for the male bones. Of note though, the previous study lacked the proof that cortical bone accrual led to an increased mechanical strength. Importantly, they do not contradict our observation that females require a more vigorous training before the cortical bone responds to the strain.

It remains to be investigated, whether the high intensity training was too strenuous for the males. We rule out the possibility that our mice lacked calcium or Vitamin D, as all animals received the same diet as breeding females in our colony that deliver normal offspring. Furthermore, we did not see any reduction in weight gain when comparing the high intensity trained mice to either non-trained controls or mice that were trained at intermediate intensity. There also was no negative correlation between bone parameters and the individual exertion measured in peak velocities as percentages of V_max_ (data not shown). Independent of whether the training was too strenuous, we tend to rule out our hypothesis, that a high cardiovascular strain per se will lead to a significantly increased bone mass.

Interestingly, the females at all times ran faster than the males and this observation has been confirmed for C57BL/6 mice, as well^13^. This difference in running speeds does not seem to correlate with weight alone, as our STR/ort females were at all times lighter than the males yet during their training period gained weight and became faster. We consider it possible that the females have different running kinematics altogether which may explain not only their lack of response to the treadmill but may protect them from osteoarthritis.

We are confident that by analyzing the hind-limbs we focused on the most promising skeletal site. It was recently reported that the hind-limbs respond better to treadmill running and show focal enhancement of the bone even though the fore-legs experience higher external forces^17^. Likewise, the vertebral bodies in quadrupedal animals require longer training periods than the limbs before they respond at all to any running exercise^18^. So why then did our mice lack any significant bone accrual and increase in mechanical strength despite a vigorous training regimen? First of all, we cannot formally rule out that the different genetic backgrounds between STR/ort and the previously used C57BL/6 or Hsd:ICR mice impact on the responses to exercise^12^,^13^,^17^. However, we would like to propose as an alternative that exercise modalities play a role. The length of the training sessions in our study was 30 minutes each and was thus identical to the previously published ones. Likewise, our training period of eight weeks was within the time frames of three to ten weeks published before^12^,^13^,^17^. Our transition from four weeks of increased frequency to four weeks of reduced frequency training followed a protocol whereby continued exercise at three weekly training sessions sufficed to maintain the benefits from the preceding increased frequency training period^19^. We therefore rule out that previously gained benefits had been lost by the time the animals were sacrificed. Indeed, we did analyze some mice after completion of only four weeks of increased frequency training and they as well did not show any bone growth (data not shown).

Our training clearly differed from previous studies in intensity. In the previous studies mice ran very slowly at constant speeds that corresponded to our active recovery periods. In contrast, peak velocities during our high intensity sprint interval trainings were as high as 25 m/min for the males and 36 m/min for the females and aimed at exceeding the lactate threshold. While we ourselves did neither measure lactate levels nor oxygen uptake, others have shown that running speeds of 34 m/min in female and 34.8 m/min in male mice correspond to maximum oxygen uptake (VO_2max_)^20^. It was further shown that training intensities within 65–85% of VO_2max_ coincide with the lactate threshold^21^ and exceeding the lactate threshold has been correlated with elevated levels of serum growth hormone which in turn stimulates bone growth^22^,^23^. Along these lines, anaerobic training has previously been described to accelerate bone turnover by increasing serum levels of bone alkaline phosphatase as well as osteocalcin and urinary pyridinoline concentrations^24^. However, there is also an exercise-dependent secretion of IGF-1, sclerostin and glucocorticoids that have opposing effects on the bone^25^. Moreover, training related inflammation and the resulting cytokines needs to be considered. While the reduced cross sectional area associated with the high intensity training in the male femora predicts a reduced moment of inertia and may thus explain the reduced mechanical strength, it appears as if the age related modelling has been halted: Bone apposition does not occur at the periosteal but at endosteal surfaces instead. This male-specific lack of periosteal bone apposition is reminiscent of a diminished IL-6/gp130 signaling that was recently suggested to be responsible for reduced periosteal bone formation in males^26^. It therefore remains to be determined to what extend our training modalities alter the serum levels of osteo-anabolic and osteo-catabolic factors. Moreover, we will specifically need to address any intra-cortical remodeling as micro-damages and stress fractures may have occurred during high intensity sprint interval trainings.

Finally, we believe it feasible, that there are limits to the peak bone mass in early adulthood and these may be genetically determined. While this hypothesis is difficult to prove in humans, we would here like to mention the woodpecker crania. If every bout of tree drumming was to induce progressive thickening, then this could constrict tiny foramina and damage nerves and blood vessels^17^. It is therefore vital that there are limits to bone mass, even though they are likely to differ between the various skeletal sites. Interestingly, the most dramatic benefits from treadmill training in rodents can be observed in young animals that had previously experienced a castration-induced bone loss^18^,^27^. It is therefore possible, that treadmill training in castrated animals helps to regain the target value of peak bone mass. Likewise, the well balanced chow and the possibility to roam ad libitum may suffice for our mice to accrue maximum peak bone mass. As our tilting of the treadmill had no effect under high intensity training conditions, it remains to be investigated whether alternative strains, resulting for instance from artificial loading, can induce bone accrual where treadmill running had no effect^25^,^28^. Finally, there are limits to our study and these concern the sample sizes. Our power analysis showed that groups as small as 4 suffice to confirm large effects but that small effects may be left undetected.

In summary, we here show that sprint interval training tailored to the individual performance limits does not seem suitable to increase peak bone mass at early adulthood. Our explanation for this negative result is threefold (i) we simply did not provide sufficient high-impact or axial loading and by increasing the decline of the treadmill we would indeed stimulate bone accrual, (ii) high cardio-vascular strains lead to the accumulation of serum factors that may be beneficial for muscle development and endurance but may restrict bone growth and iii) there are physiological limits to the peak bone mass which cannot be overcome by voluntary exercise. Translating these results to human adolescents does by no means encourage a sedentary lifestyle. It does however question whether repeated sports participation at high cardiovascular strains is supportive of maximal peak bone mass.

## Materials and Methods

### Mice

STR/ort mice were originally purchased from Harlan Winkelmann (Borchen, Germany). They were subsequently bred in our animal care facility under specific germfree conditions, housed in cages with a 12 hours light/dark cycle and given water and food ad libitum. The local state’s animal care committee (Landesamt für Landwirtschaft, Lebensmittelsicherheit und Fischerei M-V; www.lallf.de) approved all experiments (7221.3-1.1-050/13) and all experiments were carried out in accordance with the relevant guidelines and regulations.

### Treadmill training

Treadmill training was strictly voluntary meaning that, if a mouse chose not to or could not run as fast as the belt, it did not experience any punishment as for instance in the form of an electric pulse but was swept onto a platform behind the running belt. Mice (10 females, 30 males) were recruited for the treadmill (Process Control Treadmill, TSE Systems, Germany) at the age of six to seven weeks. The first two weeks served to familiarize the animals with the treadmill. This process was standardized as follows: day 1, the mice were allowed to roam freely on the resting treadmill for 10 minutes. Day 3, the mice had to run at a speed of 0.15 m/s for 10 minutes. Day 5, the speed was 0.2 m/s for 15 minutes. Day 8, the mice ran at a speed of 0.2 m/s for 10 minutes before the speed was increased to 0.25 m/s for 5 minutes. Day 10, the mice did a ten-minutes-run at 0.2 m/s before the speed was increased to 0.25 m/s for 5 minutes and again increased to 0.3 m/s for another 5 minutes. All other days were without any treadmill contact. On day 12, the mice completed the first run-to-exhaustion test (R-T-E) as shown in Fig. 1. This R-T-E served to identify the individual V_max_, defined as the maximum speed each mouse was capable of running voluntarily. Its results were needed to tailor the training program to the individual performance limits.

#### Training program

Training programs were of either high or intermediate intensity. In general, the first training period comprised four weeks of increased frequency with 5 training sessions per week followed by a second R-T-E to determine whether the mice had adapted to the training (Fig. 1). The second training period comprised four weeks of reduced frequency with 3 weekly trainings, whereby the speeds were adjusted to the results from the second R-T-E. The 24 male mice that participated in the high intensity sprint interval training were randomly divided into three groups that ran at either 10° incline, 10° decline or even. All mice were sacrificed within 30 minutes after the last training. Completely untrained 16–17 weeks old STR/ort females (n = 12) and males (n = 13) served as controls. Allocation to either control, intermediate or high intensity training groups were random. Mice were weighed every week.

#### Run-To-Exhaustion-test

The test followed a ramp up protocol slightly modified from Ingalls *et al*.^29^: after a short warming up at a speed of 10.2 m/min for 3 minutes, the speed was increased over the next 2 minutes to 15 m/min. After another two minutes - and then again every three minutes - the speed was increased in steps of 3 m/min (Fig. 1). The test was terminated when the animals no longer kept pace with the treadmill belt and was swept onto the platform behind the running belt.

#### Sprint interval training

In order to design a training that provided high magnitude strains but was not necessarily long in duration, we opted for a sprint interval training and differentiated between high and intermediate intensity. Each training session, independent of high or intermediate intensity, lasted 30 minutes and started out with a six minutes warming up period at active recovery speed. This active recovery aimed at 30–40% of the individual V_max_ achieved during the preceding R-T-E. Active recovery was followed by four bouts of 1.5-minutes intermediately or highly intense running, the former aiming at a peak velocity (V_peak_) of 60% of the V_max_ reached during the R-T-E. The 1.5-minutes bouts of high intensity running aimed at the highest possible speed the mice would run voluntarily, but no less than 75% of the V_max_. These bouts of intermediately or highly intense running were interspersed by 3.5 minutes of active recovery plus half a minute each for acceleration and deceleration (Fig. 1).

For practical reasons, treadmill training was performed in groups of four mice maximum. Allocation to these groups was based on sex and the results of the preceding R-T-E, so that homogeneous groups not only in terms of sex but also in performance were obtained. There was no drop out because an animal refused to run at all.

### X-ray micro-computed tomography (μCT)

All mice were sacrificed by cervical dislocation and the hindlegs were carefully dissected. Left hindlegs were fixed in 4% buffered paraformaldehyde (PFA) for subsequent μCT analysis. The right femora were stored for 3-point-bending tests.

After fixation in 4% PFA for 1 week, the left hindlegs were scanned in 2 ml 0.9% NaCl via μCT (SkyScan model 1076, Skyscan, Antwerp, Belgium, SN = 09H02066, Software Version 4.2). The scans were performed with an isotropic voxel size of 9 μm at 49 kV and 200 μA, a rotation step of 0.5 degree and an averaging frame of 3 was set. Reconstructions were performed with NRecon (Version 1.6.6.0), Regions of interest were set with CT-Analyzer (Version 1.12.4.0) and 2D and 3D calculations were done with BatMan (BatMan 64) as previously described^16^. In short, femoral length was determined between the femoral head (upper reference level) and the distal metaphyseal growth plate (GP) where the first crossing of the low density cartilage structure by bone primary spongiosa was set as the lower reference level. Tibial length was defined between the proximal epiphyseal growth plate (upper reference level) and the tibia-fibular syndesmosis (lower reference level). 10% each of femoral and tibial length at mid-diaphysis were analysed for cortical bone morphometry by determining the cortical bone area fractions (Bone Area/Tissue Area = B.Ar/T.Ar) and cross sectional thicknesses (Ct.Th.). Trabecular bone analyses within the femoral metaphyses started 5% above the lower reference level and comprised 10% of the total femoral length. For the tibial metaphyses, trabecular bone analyses started 5% below the upper reference level and again comprised 10% of the total tibial length. Trabecular bone phenotypes were assessed by analyzing the bone volume fraction (Bone Volume/Tissue Volume = BV/TV), trabecular thickness (Tb.Th.), trabecular numbers (Tr.N.) and structure model index (SMI). The high bone mass in STR/ort mice necessitates a clear delineation of trabecular from cortical bone. ROIs were therefore defined as circles in the centre of each image cross section that were consistent with 50% of the outer femoral and tibial diameters, respectively.

### Biomechanical analysis

After careful preparation of the bones, right femora were wrapped in gauze moistened with 0, 9% NaCl and stored in a tube at −20 °C until usage for biomechanical analysis. Bone stiffness was measured with a bending test using a 3-point bending device (Mini-Zwick Z 2.5, Zwick GmbH, Ulm, Germany). To that extent, femora were placed at a span of 0.6 cm. The loading point was placed in mid-diaphysis and the bending force was gradually increased in an anterior-posterior direction with 1 mm/min. Bending stiffness (N/mm) as well as the ultimate strength (N) were calculated from the linear elastic part of the load-displacement diagram using the software testXpert (Zwick, Version 12.0). All experiments were performed by the same examiner ensuring an identical positioning of the bones.

### Serum concentrations of RANKL and PINP

Blood serum was analyzed for Receptor Activator of NF-κB Ligand (RANKL) and Procollagen I N-Terminal Propeptide (PINP) concentrations using commercially available ELISA kits (Life Science Inc. USCN Wuhan, China) according to the manufacturer’s instructions.

### Statistics

Statistical analysis was performed in either GraphPad InStat 3 (GraphPad Software, CA, USA) or IBM SPSS Statistics 22 (IBM, NY, USA). Data were tested for normality using the Kolmogorov-Smirnov test. One-Way Anova were performed to compare groups (Figs 2, 3 and 5 and Tables 1 and 2). Two-Way ANOVA were performed to assess the possible interaction between sex and training on the various bone parameters (Tables 1 and 2). Corrections for multiple comparisons were performed according to Bonferroni. Power analysis was performed in GPower 3.1. We used previous publications to approximate the size of the expected effect^12^,^13^. Assuming large effects, an alpha error of 0.05 and a power of 0.8 yielded group sizes as small as n = 4 to reach statistical significance.

## Additional Information

**How to cite this article:** Koenen, K. *et al*. Sprint Interval Training Induces A Sexual Dimorphism but does not Improve Peak Bone Mass in Young and Healthy Mice. *Sci. Rep.* **7**, 44047; doi: 10.1038/srep44047 (2017).

**Publisher's note:** Springer Nature remains neutral with regard to jurisdictional claims in published maps and institutional affiliations.

## Supplementary Material

Supplementary Table 1

## Figures and Tables

**Table 1:** Cortical Bone Parameters in Response to the Sprint Interval Training (SIT).

	Femora			Tibiae		
	Mean ± SEM	One-way ANOVA^a^	Two-way ANOVA^b^	Mean ± SEM	One-way ANOVA^a^	Two-way ANOVA^b^
**B.Ar./T.Ar.**						
**male**						
Controls	65.6 ± 1.4	**0.0240**	n.s.	73.6 ± 1.4	**<0.0001**	n.s.
Inter. intensity SIT	71.6 ± 2.5^*^			75.0 ± 2.0^###^		
High intensity SIT	69.9 ± 0.8*			63.7 ± 0.6***		
**Female**						
Controls	78.8 ± 2.6	n.s.		87.9 ± 1.4	n.s.	
Inter. intensity SIT	87.1 ± 2.2			88.4 ± 1.4		
High intensity SIT	89.5 ± 1.5			n.d.		
**B.Ar.**						
**male**						
Controls	1.54 ± 0.05	n.s.	n.s.	1.49 ± 0.05	n.s.	n.s.
Inter. intensity SIT	1.64 ± 0.06			1.37 ± 0.02		
High intensity SIT	1.59 ± 0.03			1.39 ± 0.02		
**female**						
Controls	1.73 ± 0.05	n.s.		1.63 ± 0.04	n.s.	
Inter. intensity SIT	1.64 ± 0.06			1.60 ± 0.03		
High intensity SIT	1.59 ± 0.03			n.d.		
**T.Ar.**						
**male**						
Controls	2.35 ± 0.06	n.s.	n.s.	2.02 ± 0.05	0.0032	n.s.
Inter. intensity SIT	2.29 ± 0.10			1.84 ± 0.06^###^		
High intensity SIT	2.28 ± 0.04			2.19 ± 0.04*		
**female**						
Controls	2.20 ± 0.08	n.s.		1.81 ± 0.03	n.s.	
Inter. intensity SIT	2.08 ± 0.07			1.81 ± 0.03		
High intensity SIT	2.08 ± 0.06			n.d.		
**Ct.Th.**						
**male**						
Controls	0.35 ± 0.007	n.s.	0.056	0.32 ± 0.006	**0.0032**	n.s.
Inter. intensity SIT	0.38 ± 0.011			0.30 ± 0.007		
High intensity SIT	0.37 ± 0.006			0.2 ± 0.002***		
**female**						
Controls	0.50 ± 0.025	n.s.		0.40 ± 0.025	n.s.	
Int. intensity SIT	0.55 ± 0.046			0.42 ± 0.019		
High intensity SIT	0.61 ± 0.013			n.d.		

^a^ONE WAY ANOVA compares the means between the various training groups and investigates the impact of the training on a given parameter; ^b^TWO WAY ANOVA investigates the possible interaction between sex and training for a given parameter. All P-values were corrected for multiple comparisons. Main factors were sex and high vs intermediate training intensity. ^*^comparison to controls is statistically significant, ^#^comparison to high intensity SIT is statistically significant. Significance levels are: one symbol: P < 0.05; two symbols: P < 0.01; three symbols: P < 0.001.

**Table 2:** Trabecular Bone Parameters in Response to the Sprint Interval Training (SIT).

	**Femora**			**Tibiae**		
	Mean ± SEM	One-way ANOVA^a^	Two-way ANOVA^b^	Mean ± SEM	One-way ANOVA^a^	Two-way ANOVA^b^
**BV/TV**						
**male**						
Controls	11.0 ± 1.6	**0.0010**	n.s.	32.1 ± 5.9	n.s.	n.s.
Inter. intensity SIT	14.8 ± 2.2^###^			14.9 ± 5.6		
High intensity SIT	5.7 ± 0.6**			27.4 ± 1.9		
**female**						
Controls	35.0 ± 5.0	n.s.		41.2 ± 3.2	n.s.	
Inter. intensity SIT	29.4 ± 10.5			41.6 ± 13.2		
High intensity SIT	38.8 ± 5.9			n.d.		
**Tb.Th.**						
**male**						
Controls	0.085 ± 0.003	**0.0007**	n.s.	0.117 ± 0.011	n.s.	n.s.
Inter. intensity SIT	0.088 ± 0.005^###^			0.076 ± 0.005		
High intensity SIT	0.072 ± 0.002***			0.115 ± 0.006		
**female**						
Controls	0.09 ± 0.005	n.s.		0.117 ± 0.007	n.s.	
Inter. intensity SIT	0.009 ± 0.014			0.117 ± 0.016		
High intensity SIT	0.15 ± 0.006			n.d.		
**T.Ar.**						
**male**						
Controls	1.26 ± 0.18	**0.0064**	n.s.	2.56 ± 0.38	n.s.	n.s.
Inter. intensity SIT	1.69 ± 0.24^##^			1.93 ± 0.68		
High intensity SIT	0.78 ± 0.08*			2.38 ± 0.09		
**female**						
Controls	3.46 ± 0.36	n.s.		3.54 ± 0.30	n.s.	
Inter. intensity SIT	2.77 ± 0.59			3.34 ± 0.59		
High intensity SIT	3.61 ± 0.34			n.d.		
**Ct.Th.**						
**male**						
Controls	2.28 ± 0.1	n.s.	n.s.	1.57 ± 0.24	n.s.	n.s.
Inter. intensity SIT	2.21 ± 0.15^##^			2.25 ± 0.24		
High intensity SIT	2.67 ± 0.04***			1.47 ± 0.07		
**female**						
Controls	1.32 ± 0.22	n.s.		1.25 ± 0.25	n.s.	
Int. intensity SIT	1.57 ± 0.49			0.80 ± 0.95		
High intensity SIT	1.22 ± 0.25			n.d.		

^a^One-Way ANOVA compares the means between the various training groups and investigates the impact of the training on a given parameter; ^b^TWO WAY ANOVA investigates the possible interaction between sex and training for a given parameter. All P-values were corrected for multiple comparisons. Main factors were sex and high vs intermediate training intensity. ^*^comparison to controls is statistically significant, ^#^comparison to high intensity SIT is statistically significant. Significance levels are: one symbol: P < 0.05; two symbols: P < 0.01; three symbols: P < 0.001.
