# Supplementary material for: Sprint Interval Training Induces A Sexual Dimorphism but does not Improve Peak Bone Mass in Young and Healthy Mice
Source: Sci Rep. 2017 Mar 17;7:44047. doi: 10.1038/srep44047 (PMC5355982; doi:10.1038/srep44047)
Supplement: Supplementary Table 1 [file srep44047-s1.doc]

SPRINT INTERVAL TRAINING INDUCES A SEXUAL DIMORPHISM BUT DOES NOT IMPROVE PEAK BONE MASS in YOUNG AND HEALTHY MICE

Kathrin Koenen^1^, Isabell Knepper^1^, Madlen Klodt^1^, Anja Osterberg^1^, Ioannis Stratos^2^ Thomas Mittlmeier^2^, Tina Histing^3^, Michael D. Menger^3^, Brigitte Vollmar^4^, Sven Bruhn^5^ & Brigitte Müller-Hilke^1^*

Suppl. Table 1. Comparison of running speeds and trabecular and cortical bone parameters (femora) following high intensity interval training at different inclinations

Vmax Micro-CT

training slope mouse-ID RTE 1 RTE 2 BV/TV Tb.Th Tb.N SMI B.Ar/T.Ar B.Ar. T.Ar. Ct.Th

[m/min] [m/min] [%] [mm] [1/mm] [%] [mm^2^] [mm2] [mm]

none 1 36 21 4,9756 0,0693 0,7177 2,6841 65,8730 1,4416 2,1884 0,3462

none 2 30 27 3,4025 0,0644 0,5287 2,7892 71,9791 1,5479 2,1505 0,3754

none 3 39 39 6,2870 0,0836 0,7521 2,5784 69,4274 1,7314 2,4939 0,3898

none 4 39 27 9,4637 0,0852 1,1103 2,3791 64,8708 1,4280 2,2013 0,3350

none 5 39 30 14,6344 0,0864 1,6939 2,2147 71,6763 1,4586 2,0349 0,3549

none 6 n.d. n.d. 4,5889 0,0836 0,5490 2,7662 56,5533 1,3074 2,3118 0,3137

none 7 n.d. n.d. 8,5958 0,0893 0,9629 2,0295 58,3768 1,4896 2,5517 0,3320

none 8 n.d. n.d. 8,7270 0,0813 1,0739 2,2096 57,7447 1,4461 2,5043 0,3320

none 9 n.d. n.d. 14,0470 0,0907 1,5481 1,8496 68,1956 1,7306 2,5377 0,3219

high intensity down 10° 1 27 24 6,0021 0,0728 0,8249 2,5853 73,6078 1,2301 2,0521 0,4132

high intensity down 10° 2 36 36 1,4395 0,0649 0,2219 2,8070 65,5615 1,6681 2,2662 0,3474

high intensity down 10° 3 39 42 1,8974 0,0633 0,2999 2,7257 66,2227 1,6717 2,4495 0,3461

high intensity down 10° 4 30 42 3,9858 0,0865 0,4609 2,7552 68,6889 1,8515 2,5180 0,3560

high intensity down 10° 5 30 30 2,6557 0,0608 0,4369 2,7991 68,2466 1,4554 2,2199 0,3486

high intensity down 10° 6 27 36 5,2813 0,0758 0,6964 2,3638 59,9435 1,4865 2,2447 0,2984

high intensity down 10° 7 33 30 9,9234 0,0889 1,1159 2,7962 73,5306 1,5251 2,2203 0,4096

high intensity down 10° 8 27 36 1,7435 0,0695 0,2508 3,0884 68,4037 1,8075 2,6424 0,3880

high intensity none 1 27 27 5,6226 0,0696 0,8077 2,6371 64,5361 1,4196 2,1997 0,3420

high intensity none 2 39 30 6,6312 0,0688 0,9638 2,7045 66,9896 1,7288 2,5807 0,3841

high intensity none 3 33 33 10,7920 0,0822 1,3123 2,5080 76,7603 1,8064 2,3533 0,4205

high intensity none 4 39 33 7,9596 0,0723 1,1005 2,5246 72,4590 1,7259 2,3819 0,3751

high intensity none 5 33 30 4,8262 0,0652 0,7400 2,7296 72,8587 1,4818 2,0338 0,3604

high intensity none 6 27 27 2,9001 0,0717 0,4048 2,7909 66,8780 1,4768 2,2082 0,3468

high intensity none 7 33 36 4,0804 0,0776 0,5261 2,7619 73,4417 1,8934 2,5781 0,3915

high intensity none 8 30 21 10,4730 0,0725 1,4451 2,3567 72,9773 1,6876 2,3125 0,3833

high intensity up 10° 1 30 36 9,8917 0,0687 1,4405 2,4099 73,6742 1,6657 2,2609 0,3816

high intensity up 10° 2 30 33 2,9376 0,0583 0,5043 2,6241 67,3475 1,2761 1,8948 0,3237

high intensity up 10° 3 30 33 2,8237 0,0638 0,4425 2,8197 72,9525 1,5250 2,0904 0,3715

high intensity up 10° 4 33 33 4,6739 0,0666 0,7018 2,5849 69,7992 1,4498 2,0771 0,3540

high intensity up 10° 5 27 30 6,9508 0,0738 0,9413 2,7320 73,2803 1,6491 2,2504 0,3771

high intensity up 10° 6 27 30 6,8790 0,0851 0,8085 2,7327 69,3368 1,5295 2,2059 0,3608

high intensity up 10° 7 27 30 6,6912 0,0725 0,9235 2,7709 70,7268 1,5881 2,2454 0,3782

high intensity up 10° 8 30 33 9,6155 0,0707 1,3601 2,3700 68,9116 1,6259 2,3594 0,3839

ONE WAY ANOVA: 0,0835 0,0390 0,0816 0,0274 0,0353 0,3179 0,2376 0,1587
